# Self-Administered Six-Minute Walk Test Using a Free Smartphone App in Asymptomatic Adults: Reliability and Reproducibility

**DOI:** 10.3390/ijerph19031118

**Published:** 2022-01-20

**Authors:** Matheus Oliveira de Jesus, Thatiane Lopes Valentim Di Paschoale Ostolin, Neli Leite Proença, Rodrigo Pereira da Silva, Victor Zuniga Dourado

**Affiliations:** 1 Department of Human Movement Sciences, Federal University of São Paulo (UNIFESP), Santos 11015-020, SP, Brazil; matheus.oliveira93@hotmail.com.br (M.O.d.J.); thatiane.ostolin@unifesp.br (T.L.V.D.P.O.); nelieluana@gmail.com (N.L.P.); r.pereirads@hotmail.com (R.P.d.S.); 2 Lown Scholars Program, Harvard T.H. Chan School of Public Health, Boston, MA 02115, USA

**Keywords:** cardiorespiratory fitness, accelerometer, 6-minute walk test, physical activity level, health and public health, app, applications

## Abstract

Background: The 6-min walk test (6MWT) is a simple, inexpensive, reliable, and reproducible test that provides a reasonable estimate of the cardiorespiratory fitness (CRF). We aimed to assess the reliability and reproducibility of a self-administered 6MWT in asymptomatic adults using a free smartphone app. Methods: In the 1st phase, 93 participants underwent a supervised 6MWT (6MWTsup) in a 30 m indoor corridor, using a triaxial accelerometer and their smartphones to compare the total step counts and to develop a 6-min walk distance (6MWD) prediction equation. In the 2nd phase, 25 participants performed the 6MWTsup and two self-administered 6MWTs outdoors (6MWTsa1 and 6MWTsa2, at least 48 h apart) using a free smartphone app. Results: The agreement between accelerometer- and app-based total step counts was limited (mean difference, −58.7 steps (−8.7%): 95% confidence interval, −326.5 (−46.8%) to 209.1 (29.3%)). The best algorithm for predicting the 6MWTsup_m_ included: 795.456 + (0.815 height_m_ app-steps) − (1.620 age_years_) − (3.005 weight_kg_) − (1.155 app-steps), R^2^ = 0.609). The intraclass correlation coefficient between 6MWTsa2 and 6MWTsa1 was excellent (0.91: 0.81–0.96). The coefficient of variation was 6.4%. The agreement between the two self-administered tests was narrow (−1.9 (0.2%) meters: −57.4 (−9.5%) to 61.3 (9.9%)). Conclusions: The self-administered 6MWT has excellent reliability and reproducibility in asymptomatic adults, being a valuable tool for assessing CRF in community-based interventions.

## 1. Introduction

Cardiorespiratory fitness (CRF) is one of the main domains of physical fitness. It refers to the cardiovascular, metabolic, and respiratory systems’ ability to absorb, transport, and consume oxygen during the long-term physical effort of moderate-to-vigorous intensity [1]. High CRF is associated with lower cardiovascular and all-cause mortality [2,3]. In contrast, low CRF is considered one of the causes of diabetes mellitus, hypertension, coronary artery disease, and several types of cancer [4]. CRF alone holds predictive value for cardiovascular events, similar to the combination of classic cardiovascular risk factors [4]. CRF evaluation is essential to predict and assess the prognosis of several diseases [5] and the prescription of physical exercise and monitoring rehabilitation programs’ results [4]. Accordingly, CRF has been considered a vital sign in cardiovascular health as a routine clinical practice strategy [4]. However, if the administration of the maximum exercise test is not possible, submaximal, and field tests—and even CRF estimation using prediction equations—must be performed in environments with few resources. Despite the evidence, the evaluation of CRF is yet to be incorporated as a routine strategy for screening cardiovascular risk.

The 6-min walk test (6MWT) is a simple, inexpensive, reliable, and reproducible test performed according to international standardization [6]. Initially, the 6MWT was developed to assess patients with cardiorespiratory diseases; however, it has already been validated in several diverse populations [7]. Furthermore, the 6MWT correlates consistently with the maximum oxygen consumption (VO^2^max) both in patients with chronic diseases and in asymptomatic adults [8], and has several reference values [7]. The test is usually performed in a controlled environment [6] and has already been performed on thousands of patients with chronic diseases without any report of serious cardiovascular events [9,10], which suggests its safety. Therefore, the 6MWT provides a reasonable estimate of the CRF, especially in people with non-severe chronic diseases.

The six-minute walk test may be helpful in healthy asymptomatic subjects for cardiovascular risk screening in the clinical practice [4], due to its low cost, easy application, and performed in international standardization [6]. Furthermore, a walking distance below 96% of the predicted values can identify asymptomatic adults with low physical activity levels [11] and cardiorespiratory fitness [12]. Thus, it can be an excellent strategy to reduce the risk of cardiovascular disease.

The use of health applications (apps) has increased dramatically with the advent of mobile technology devices, such as smartphones and tablets. Their low cost and ease of use turned them into objects of epidemiological studies aiming at behavior changes through goal-setting, self-monitoring, and performance feedback [13]. Moreover, the efficiency of apps, combined with the ability to measure the distance covered by the Global Positioning System (GPS) and the number of steps using accelerometers built into the devices, opens up the possibility of using smartphone apps to self-administer the 6MWT. Thus, self-assessment of CRF would enable better cardiovascular risk screening and information for clinicians and primary care professionals. However, to the best of our knowledge, few studies have investigated the reliability and reproducibility of self-administration of the 6MWT [14,15,16], mainly involving patients in rehabilitation who undergo routine treatment with 6MWT and using paid apps. We are unaware of previous studies investigating the reliability and reproducibility of a self-administered 6MWT in the general population using a free smartphone app with a step-monitoring function.

Accordingly, this study aimed to assess the reliability and reproducibility of a self-administered 6MWT in asymptomatic adults using a free smartphone app for step counting.

## 2. Materials and Methods

### 2.1. Sample, Recruitment, and Design

The first phase of the study comprised 93 asymptomatic participants aged between 30 and 70 years old, with non-relevant cardiorespiratory, metabolic, or locomotor disorders that could preclude normal gait, as well as physical exercise. Participants should be engaged in technology, i.e., they use smartphone devices. We recruited participants through social networks, posters at regional universities, and local print media. Participants with abnormalities found during exercise testing, which could prevent the performance of unsupervised physical exercises, were excluded, such as an exam suggestive of myocardial ischemia, potentially lethal arrhythmias, and hyper-reactive blood pressure responses. We also excluded participants with spirometric abnormalities.

This phase’s objective was to develop a 6MWT distance prediction equation using the number of steps performed and demographic and anthropometric attributes. The second phase consisted of 25 participants performing a supervised 6MWT (6MWTsup), and within the seven days after that, two more self-administered tests (6MWTsa) were performed, either indoors or outdoors, and in a safe environment, using a free smartphone app available at app stores. The study was submitted to, and approved by, the local Human Research Ethics Committee. All participants signed an informed consent form related to the study’s objectives, methods, benefits, and possible risks.

The study participation was formalized by the signature of the free and informed consent form. The research was approved by the ethic in research committee of the Federal University of Sao Paulo, CEP/UNIFESP #1485/2017.

### 2.2. Application Choice

The researchers tested several free smartphone apps before starting the study; the most popular apps with a step count function that worked on the most popular operating systems (i.e., iOS and Android) were downloaded. In this list, we found applications such as Fitbit, Apple Health, Google fit, MyFitnessPal, MapMyWalk, Runtastic, and Runkeeper, among others. After a final meeting, we chose the app Pacer, mainly based on user ratings. Pacer has 4.9 rating stars in the app store and was reported by researchers as the most attractive. The researchers agreed with this choice due to some characteristics of the app mentioned above, which are as follows: (1) its proper functioning on the two most popular operating systems; (2) its accuracy in monitoring the number of daily steps; (3) the app’s several gamification functions, such as goal setting, rewards, badges, progress bars, walking and running rankings, and forming groups with their social network. Although not necessary for the present study, these characteristics were decisive in stimulating technology to increase physical activity and fitness. Therefore, we investigated the agreement between the number of steps provided by the Pacer application and the triaxial accelerometer during the supervised six-minute walk test. We used the equation developed in the first phase to estimate the distance in the 6MWTsa performed with Pacer in the second phase.

### 2.3. Phase 1 Protocol

The phase 1 evaluation protocol was divided into two days, with a seven-day interval. On the first day of the evaluation, the participants underwent an initial health screening. After obtaining anthropometric measurements, we performed spirometry and cardiopulmonary exercise testing. On the second day, participants performed the 6MWTsup according to international recommendations [6].

### 2.4. Health Screening

We obtained from participants socio-demographic data (age, sex, schooling), health history, and classic cardiovascular risk factors for cardiovascular diseases, such as current smoking, obesity, high blood pressure, diabetes mellitus, dyslipidemia, and physical inactivity. Those who reported less than 150 min/week of moderate to intense physical activity or less than 75 min of intense physical activity per week were considered physically inactive [17]. We measured height (cm) and body mass (kg) using a digital scale with a stadiometer (Toledo™, Sao Paulo, Brazil) to calculate the body mass index (BMI) (kg/m*^2^*). Obesity was considered when BMI ≥ 30 kg/m*^2^*.

### 2.5. Supervised 6MWT

After seven days, participants performed the 6MWTsup in an indoor 30 m hallway under the supervision of two experienced examiners, following international recommendations [6]. The participants used a triaxial accelerometer (ActiGraph GTX3x+, MTI, Pensacola, FL, USA) to count the number of steps during the test. The participants simultaneously performed the 6MWTsup with their smartphones in their pocket to record the total number of steps obtained in the Pacer app. We recorded the distance covered in the 6MWT (6MWD) and the total accelerometer and app-based step counts.

### 2.6. Phase 2 Protocol

Phase two comprised 25 participants for the cross-validation sample, and participants performed a 6MWTsup in the same corridor adjacent to the laboratory, strictly equal to that performed in phase 1 for the first study sample.

### 2.7. Self-Administered 6MWT

The cross-validation sample (*n* = 25) performed two tests at least 24 h apart. We instructed participants to measure baseline measurements of heart rate, perceived exertion, and blood pressure. Heart rate was measured by a validated smartphone app [18], and dyspnea and leg fatigue using the Borg scale [19]. We advised participants to use their own devices or visit a health facility (pharmacy, hospitals, or health centers) on the day of the test to measure their blood pressure.

For the test to be performed effectively, the participants were instructed to perform the tests on a straight course and, preferably, going back and forth over 20 m, in an open place with mild temperatures around 26 °C. The participants were also allowed to perform the tests indoors, as long as the course was long enough and straight. All were instructed to perform two 6MWTs in the same climatic conditions, time of day, and course.

The cardiopulmonary exercise test results, especially of the stress electrocardiogram, were discussed previously to ensure the participants’ safety. Furthermore, four measurements of blood pressure at rest in the seven days of the protocol were performed, which allowed for rigorous screening of these participants with the cardiologist. All were advised not to perform the tests if the heart rate was above 120 bpm and/or blood pressure above 150/100 mmHg. The 6MWTsa distances were estimated based on the equation developed in phase 1 and then were compared to the 6MWTsup of phase 2.

### 2.8. Statistical Analysis

Statistical analysis was conducted using the statistical packages SPSS (IBM Corp., Armonk, NY, USA), version 23, and MedCalc (MedCalc Software Ltd., Ostend, Belgium), version 14. The data are presented as mean ± standard deviation for continuous variables, and counts/percentages for categorical variables.

#### 2.8.1. Phase 1

The initial comparison was between the accelerometer- and app-based step counts obtained in the 6MWTsup in the initial sample (*n* = 93). We compared means using Student’s *t*-test. The reliability of the app was assessed by calculating the intraclass correlation coefficient (ICC) and its 95% confidence interval (95%CI), and the coefficient of variation (CV) of the mean difference to its standard deviation. We used the Bland plots method [20] to investigate the agreement between accelerometer- and app-based step counts by calculating the mean difference and its 95%CI against the average of the two approaches.

We fit stepwise multiple linear regressions to predict the 6MWTsup distance based on the total step counts and demographic (age and sex) and anthropometric (weight, height, and BMI) variables. A sensitivity analysis was used to identify the main predictors with linear models and a quadratic model, including age × age*^2^*. The potential of the variable height × 0.41 and the interaction between height × number of steps was also investigated, as previously suggested [14]. We used the same sensitivity analysis to compare the step counts between the devices used (i.e., accelerometer vs. app). We found a stronger correlation of the distance walked with the app-based step counts, compared with that observed for the accelerometer-based counts. Because of the easier applicability of the app-based step counts in real life, the best equation was developed based on the app-based step counts. Considering an R*^2^* of this equation around 0.50 in previous studies [21], the statistical power of 80%, the alpha error of 0.05, and five predictors included in the analysis, 93 participants were needed to elaborate the equation in Phase 1 [22].

#### 2.8.2. Phase 2

In a second cross-validation sample (*n* = 25), the 6MWTsup was conducted under the same conditions to obtain the measured 6MWTsup distance. The Pacer app was used to evaluate the measurement properties of the 6MWTsa. The first and second 6MWTsa distances were estimated using the prediction equation developed in phase 1. We compared the distances using the paired Student’s *t*-test. The reliability of the 6MWTsa was investigated by the ICC and 95%CI and by the CV. The reproducibility of the 6MWTsa was evaluated by the Bland and Altman plot [23] comparing the second 6MWTsa (6MWTsa2) and first 6MWTsa (6MWTsa1). We also investigated the agreement between 6MWTsa2 and 6MWTsup and 6MWTsa1 and 6MWTsup using the Bland and Altman method.

We considered the ICC values smaller than 0.5, between 0.5 and 0.75, between 0.75 and 0.9, and greater than 0.90 as indicative of weak, moderate, good, and excellent reliability, respectively [20]. For CV, the values considered acceptable were lower than 12%. We set the probability of alpha error for all tests at 5%.

## 3. Results

We evaluated 93 participants during phase 1 (39 men, 54 women). The participants were, on average, middle-aged and overweight. Except for a higher proportion of obese subjects and a slightly lower proportion of smokers, the other cardiovascular risk factors remained similar to the general population in Brazil [24] (Table 1). Phase 2’s 25 participants were significantly younger, predominantly male, and represented a lower proportion of hypertensive and diabetic patients. However, they presented higher smoking and physical inactivity ratios (Table 2).

The distance covered in the 6MWTsup was significantly correlated with age, height, BMI, accelerometer-based step counts, and app-based step counts. Although it did not reach statistical significance, weight presented a weak correlation with 6MWTsup (Table 3). After stepwise multiple regression analysis, the interaction between height and app-based step counts, age, weight, and app-based step counts was the main predictor of 6MWDsup, explaining almost 61% of outcome variability (Table 4).

**Figure 1:**
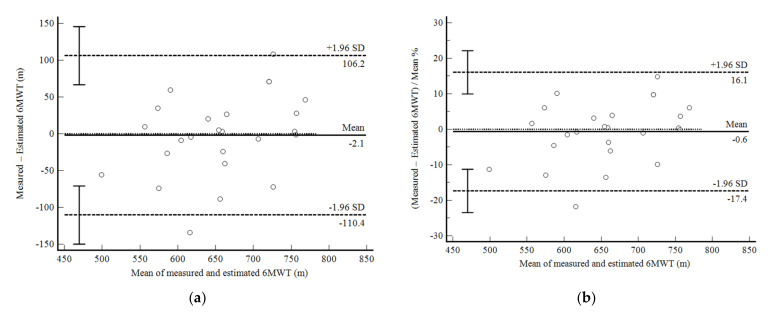
Bland and Altman graphic method with limits of agreement and 95% confidence interval (95%CI) between the measured and estimated six-minute walk test (6MWT) using the equation developed in the present study (Phase 1). (**a**) absolute values; (**b**) percentage values.

The mean difference between measured and estimated 6MWD calculated using the equation developed in phase 1 was only −2.1 m (0.6%) with a 95% confidence interval (95%CI) from −110.4 (−17.4%) to 106.2 (16.1%) (Figure 1a,b). The correlation between values was strong (r = 0.77; *p* < 0.001; R^2^ = 0.59). The ICC was good (0.855: 0.671–0.936), as was the CV (6.1%). Although it did not reach statistical significance, the mean values of 6MWD tended to be positively correlated with the mean difference (*p* = 0.07).

**Figure 2:**
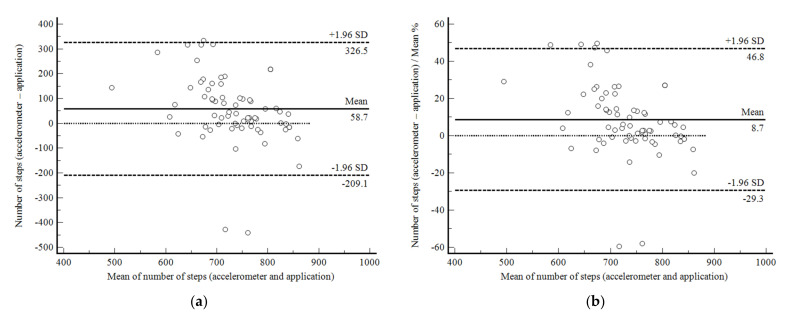
Bland and Altman graphic method with limits of agreement and 95% confidence interval (95%CI) between the accelerometer- and app-based number of steps during the supervised six-minute walk test in 93 subjects enrolled in Phase 1 of the present study. (**a**) absolute values; (**b**) percentage values.

The agreement between accelerometer- and app-based total step counts during the 6MWTsup was limited (mean difference, −58.7 steps (−8.7%): 95% confidence interval, −326.5 (−46.8%) to 209.1 (29.3%)) (Figure 2a,b). The correlation between the two measurements was non-significant (r = 0.13; *p* = 0.832) and the CV was higher than 12% (16.4%).

**Figure 3:**
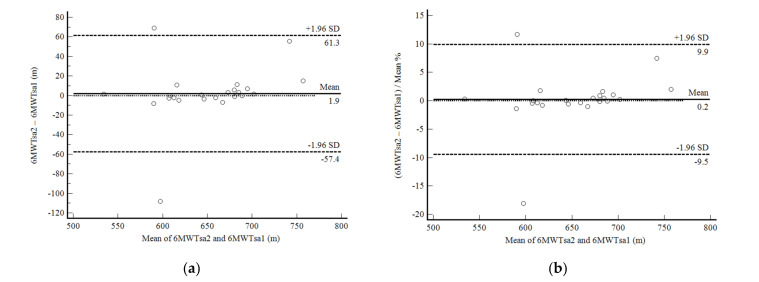
Bland and Altman graphic method with limits of agreement and 95% confidence interval (95%CI) between the distance walked estimated by the number of steps and demographic and anthropometric attributes in the second self-administered six-minute walk test (6MWTsa2) and first (6MWTsa1). (**a**) absolute values; (**b**) percentage values.

In phase 2 of the study, the self-administered 6MWD showed excellent reproducibility. The 6MWTsa2 was less than two meters different from the 6MWTsa1 (mean difference, 1.94 ± 30.27 m; *p* 0.761), and the difference was normally distributed. Confidence limits were shallow in 22 of 25 participants and were less than 10% (Figure 3a,b). The correlation between the mean difference and the mean values was not significant. Reliability between measurements was also excellent (ICC, 0.91: 95%CI, 0.81–0.96; CV = 6.4%).

**Figure 4:**
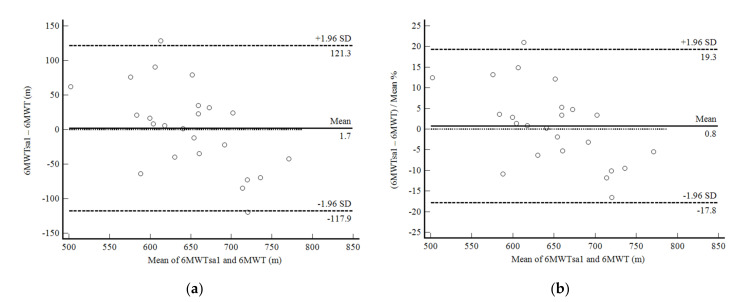
Bland and Altman graphic method with limits of agreement and 95% confidence interval (95%CI) between the distance walked estimated by the number of steps and demographic and anthropometric attributes in the first self-administered six-minute walk test (6MWTsa1) and the supervised six-minute walk test distance (6MWT). (**a**) absolute values; (**b**) percentage values.

The 6MWTsa1 was not significantly different from the 6MWTsup (mean difference, 1.68 ± 61.00 m; *p* = 0.894). The reliability of this evaluation was moderate (ICC, 0.75: 95%CI, 0.42–0.89; CV = 27.5%). The confidence limits were acceptable around 19%, but with a significant negative correlation between the mean difference and the mean values, i.e., the 6MWDsa1 overestimated the results of the subjects who took fewer steps and underestimated the ones who took more steps (Figure 4a,b).

**Figure 5:**
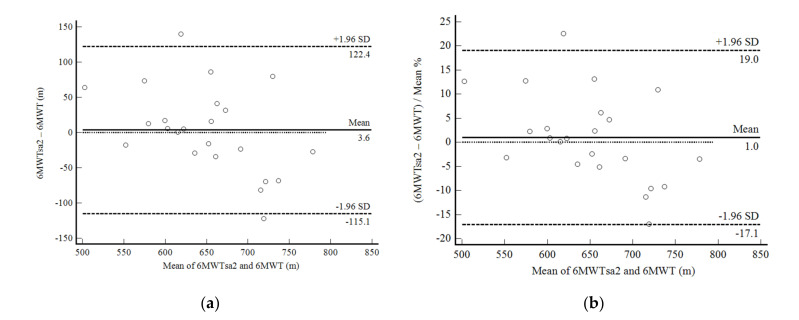
Bland and Altman graphic method with limits of agreement and 95% confidence interval (95%CI) between the distance walked estimated by the number of steps and demographic and anthropometric attributes in the second self-administered six-minute walk test (6MWTsa2) and the supervised six-minute walk test distance (6MWT). (**a**) absolute values; (**b**) percentage values.

We observed similar results for 6MWDsa2 as above mentioned. Although the mean difference was not statistically significant (3.64 ± 60.57 m; *p* = 0.776), reliability was considered good (ICC, 0.79: 95%CI, 0.50–0.91; CV = 6.0%). Confidence limits of agreement were acceptable, lower than 19%, but with a significant negative correlation similar to that described for the 6MWTsa1 (Figure 5a,b).

## 4. Discussion

This study showed the excellent reliability and reproducibility of the self-administered 6MWT in asymptomatic adults using a free-of-charge smartphone app. Although the results were less consistent, the self-administered tests also managed to estimate the accurate distance covered in the 6MWT in a wide range of physical fitness without significant differences. These results were observed despite the low accuracy of the number of steps quantified by the smartphone concerning the direct reference method, i.e., triaxial accelerometer. Nevertheless, demographic, anthropometric attributes, and the interaction between height and app-based step counts ensured greater validity of the proposed estimates.

The main finding was the excellent reliability and reproducibility of 6MWDsa2 with 6MWDsa1. Reliability measures (ICC = 0.91: 0.81–0.96; CV = 6.4%) were excellent and the confidence limits were narrow, below 10% bias. Our results for asymptomatic adults are even more consistent than those previously described for heart failure patients [14] and cancer [15]. Brooks et al. [14] evaluated patients with heart failure in a very similar design to our study. The reliability of the measurements was also high (ICC = 0.88, 95% = 0.77–0.98, IC = 4.7%) after performing the self-administered 6MWT per week (3.2 ± 1.0). The mean difference between the distances was only 7.6 ± 26 m and was less than 15% in 100% of the patients evaluated in Brooks et al. [14]. Through a similar equation and involving the interaction between height and app-based step counts, we observed a mean difference of only 1.94 ± 30.27 m, less than 10% in 96% of cases. These results are encouraging. Our study also presents the advantage of a free app available in the two largest app stores, with good functionality in both iOS and Android systems. We were unable to find the app SA-6MWT to be downloaded, which is the one used in the study by Brooks et al. [14]. Douma et al. [15] used an app also available free of charge (Walkmeter) for self-administration of 6WMT. Reliability values were adequate (ICC = 0.88: 0.74–0.94), unlike the limits of agreement that were limited; lower agreement can be attributed to the fact that the distance traveled in the self-administered tests was quantified with GPS signal since the used app does not have measurements of the number of steps. The results indicate that the self-administration of the 6MWT is promising in patients and asymptomatic adults and that the number of steps, combined with demographic and anthropometric attributes, can improve the effectiveness of this strategy [25].

Regarding the reliability and agreement of 6MWDsa with 6MWDsup, our results were less consistent than those mentioned above. However, the equation developed in our study was able to estimate the 6MWDsa without statistically significant differences for 6MWDsup. The 6MWDsa1 was only 1.68 ± 61.00 m higher than 6MWDsup, while the 6MWDsa1 was 3.64 ± 60.57 m higher. The reliability of the 6MWDsa2 was also good (ICC, 0.79: 95%CI, 0.50–0.91; CV = 6.0%). The limits of agreement were lower than 20%. The results of Brooks et al. [14] were similar to 91% of patients showing differences of less than 15%. These limits of reliability are within what is considered acceptable. Again, the use of GPS in the study by Douma et al. [15] in cancer patients showed limitations to the actual estimation of 6MWD in supervised testing. However, both in our study and Brooks et al. [14], there was a correlation between the mean difference and the mean values of the 6MWD, indicating bias in physical fitness extremes. Our results indicate that the equation developed underestimates the real 6MWD, especially in those with a higher CRF level who took more steps during the 6MWT. Similar results were described for another physical activity monitor, with overestimation at lower walking speeds and underestimation at higher speeds [26]. Our equation should thus be used with caution in fitter individuals.

Our results suggest that the 6MWDsa assessed in this study is very precise, considering its excellent reproducibility; however, its accuracy is limited, especially in individuals with more significant physical fitness. However, we can suggest the great practical applicability of our results. High CRF is a vital health index [2,3], and the VO2max measurement is the only index capable of increasing the predictive power of cardiovascular risk scores containing classic risk factors [4]. Adding CRF to traditional risk factors means reclassifying the risk of poor health outcomes. Even globally used and recognized cardiovascular risk scores such as Framingham’s can be improved with CRF in the prediction model [27]. The justification for the non-inclusion of CRF in cardiovascular event prediction models lies in its need for sophisticated equipment and highly trained human resources make it challenging to use on a large scale. However, clinical field tests such as the 6MWT can make this assessment more feasible in the clinical and public health environment. The literature, although still controversial, shows that VO2max can be estimated through 6MWD, both in patients with chronic diseases and in asymptomatic individuals [5,8]. Although the accuracy of the 6MWD estimate of the present study may be questioned, we still suggest that the proposed strategy is valid. If we are unable to measure VO2max, it is recommended to use submaximal tests, field tests, and even estimates without exercise [4]. The latter presents an error in the VO2max estimate around 2.98 to 6.90 mL/min/kg, similar to the error described for submaxims [28]. Regardless of the questionable accuracy of these CRF prediction equations, two of them—by Jurca et al. [29] and Nes et al. [30]—showed a reduction in the risk of long-term mortality [31,32], as well as reduction of cardiovascular risk compatible with the reduction described for the direct CRF measurement [33]. Thus, our strategy can be useful for the periodic evaluation of CRF and places it as a vital sign in the clinical environment and public health [4].

Our results indicated the app’s tendency to underestimate the actual number of steps performed (8.7%; 58.7 steps). Our results are following the previously described. Green et al. [21] compared the number of steps quantified by smartphone apps, both iOS and Android, with the direct visual measurement evaluated by video at three treadmill walking speeds (2.5, 5, and 7.7 km/h). The biases described were 9%, 5.5%, and 12.8%, respectively. There were no significant differences between the various smartphone brands. Considering that the 6MWD of asymptomatic adults varies between 450 and 790 m, which corresponds to speeds between 4.5 and 7.9 km/h, our results were very similar to those described by Green et al. [21]. The literature shows that the different apps’ algorithms influence the measurements more than the smartphone brand.

Some limitations of our study must be addressed. Our cross-validation sample presented some distinct characteristics of the sample used to elaborate the 6WMD prediction equation, due to our convenience sample. Also, we used the available sample at our convenience for the cross-validation phase. Even though we did not calculate a sample size a priori, we used the MedCalc software version 14 to calculate the mean difference and its standard deviation, and the maximum allowed difference for a sample size of 25 as in the present study. Considering the best scenario of a mean difference of zero and a standard deviation of the mean difference of 53 m in our previous experience in asymptomatic subjects [12], we found the maximum allowed difference of 170 m. This distance traveled is much larger than found in our 6MWDsa2 vs. 6MWDsa1 Bland and Altman plot (61.3 m), and even more extensive than our worse result of 122.4 m. Thus, we are confident that our limits of agreement support our conclusions. Nevertheless, our results were valid, and we believe them to be helpful in both cases. Regarding measurement properties, the responsiveness of the 6MWTsa was not evaluated in this study, as well as in previous studies. Therefore, this property, both in apparently healthy people and in patients with chronic diseases, remains unknown. Nonetheless, given the excellent responsiveness of the supervised 6MWT, including the minimum clinically relevant distance available, we can expect the 6MWT to have good responsiveness. We also performed the 6MWTsup in the 25 participants in the cross-validation section. Thus, they could have learned how to do the 6MWT correctly. However, whether our results can be applied to an utterly naïve app user remains unknown. Finally, considering the increase in cardiovascular health problems in people under 40 years of age, we decided to include younger adults between 30 and 39 years of age who presented lower cardiovascular risk and higher CRF, which may have contributed to underestimating 6MWT by the equation developed.

This study presents considerable potential. To the best of our knowledge, this is the first study to evaluate the measurement properties of the self-administered 6MWT in a sample of asymptomatic adults, which allows us to suggest the use of this strategy in primary care in CRF monitoring and health promotion programs. We also developed the equation of predicting 6MWD in a sufficient sample of individuals, which increases the internal validity of our results. Additionally, our study used a free app with optimal functionality on the two most popular smartphone operating systems, unlike the unavailable app developed by Brooks et al. [14] and the free app lacking step count monitoring as investigated by Douma et al. [15].

Our results present great practical applicability. The incremental and symptom-limited CPET requires sophisticated, expensive equipment and requires highly skilled staff. Although the incremental walk test is a field test, it has the characteristic of controlled speed, which allows exercise at high and even maximum intensity in the asymptomatic population [7]. The 6MWT, in turn, presents extensive validity, reliability, reproducibility, and responsiveness determined in the literature [6,34]. The test has been widely used to monitor the progression of functional exercise capacity and the results of several interventions [4]. Thus, the self-administration of this test would allow the self-monitoring of several morbidities and chronic diseases. Because it is a submaximal test, it has already been applied to thousands of patients with severe diseases without reports of adverse effects and non-fatal or fatal events [4]. Probably because of this, the 6MWT is less intimidating in more severe health conditions [35]. Therefore, the self-administered 6MWT is promising in achieving the AHA (American Heart Association) objectives to include periodic evaluation of CRF definitively as a vital sign in the clinical environment.

## 5. Conclusions

We can conclude that the self-administration of the 6MWT in asymptomatic adults using a smartphone app is valid and presents excellent reliability and reproducibility. This strategy may be useful for the periodic evaluation of CRF in the clinical environment and in primary care. However, our results suggest that the 6MWT should be used with caution in people with higher CRF.

## Figures and Tables

**Table 1:** General characteristics of sample enrolled in phase 1 to develop the prediction equation for six-minute walk distance (*n* = 93).

Variables	Males (*n* = 39)	Females (*n* = 54)	Total Sample (*n* = 93)
Age (years)	42 ± 6	46 ± 11	44 ± 10
Weight (kg)	81.0 ± 17.7	70.4 ± 11.8	74.8 ± 15.4
Height (cm)	173 ± 6	59 ± 6	165 ± 9
BMI (kg/m^2^)	26.8 ± 4.4	27.6 ± 5.2	27.3 ± 4.9
**Cardiovascular risk factors, *n* (%)**			
Arterial hypertension ^a^	6 (15.4)	11 (20.4)	17 (18.3)
Diabetes ^a^	2 (5.1)	8 (14.8)	10 (10.8)
Dyslipidemia ^a^	6 (15.4)	11 (20.4)	17 (18.3)
Obesity	8 (20.5)	16 (29.6)	24 (25.8)
Current smoking ^a^	3 (7.7)	7 (13)	10 (10.8)
Physical inactivity	4 (10.3)	3 (5.6)	7 (7.5)
Six-minute walk distance (m)	670 ± 65	602 ± 81	633 ± 81
Step counts (accelerometer)	767 ± 84	758 ± 76	762 ± 79
Step counts (application)	721 ± 112	686 ± 111	704 ± 112

Data were expressed as mean ± standard deviation of frequency (%). BMI: Body Mass Index. ^a^: cardiovascular risk factor self-reported.

**Table 2:** General characteristics of the sample used for cross-sectional validation (*n* = 25).

Variables	Males (*n* = 16)	Females (*n* = 9)	Total Sample (*n* = 25)
Age (years)	38 ± 8	41 ± 10	36 ± 9
Weight (kg)	88.6 ± 18.4	70.2 ± 13.5	82.0 ± 18.8
Height (cm)	176 ± 6	162 ± 5	171 ± 9
BMI (kg/m^2^)	28.4 ± 5.0	26.6 ± 5.0	27.8 ± 5.0
**Cardiovascular risk factors, *n* (%)**			
Arterial hypertension ^a^	2 (12)	0 (0)	2 (8)
Diabetes ^a^	0 (0)	0 (0)	0 (0)
Dyslipidemia ^a^	3 (18)	3 (33)	6 (24)
Obesity	3 (18)	2 (22)	5 (20)
Current smoking ^a^	3 (18)	1 (11)	4 (16)
Physical inactivity	4 (25)	5 (55)	9 (36)
Six-minute walk distance (m)	663 ± 79	640 ± 69	648 ± 84
Step counts (accelerometer)	754 ± 105	761 ± 93	754 ± 98
Step counts (application)	707 ± 112	644 ± 138	680 ± 123

Data were expressed as mean ± standard deviation of frequency (%). BMI: Body Mass Index. ^a^: cardiovascular risk factor self-reported.

**Table 3:** Correlation matrix between the distance covered in the supervised 6MWT (6MWD), demographic, anthropometric attributes and the total number of steps performed in the test (*n* = 93).

Variables		6MWD (m)	Age (years)	Height (cm)	Weight (kg)	BMI (kg/m^2^)	Accelerometer-Based Step Counts	App-Based Step Counts
6MWD (m)	r	1	−0.499 **	0.459 **	−0.166	−0.512 **	0.350 **	0.351 **
	*p*		0.000	0.000	0.073	0.000	0.000	0.003
Age (years)	r	−0.499 **	1	−0.427 **	−0.124	0.155	−0.215 *	−0.021
	*p*	0.000		0.000	0,180	0.094	0.020	0.864
Height (cm)	r	0.459 **	−0.427 **	1	0.505 **	−0.091	−0.130	0.061
	*p*	0.000	0.000		0.000	0.327	0.162	0.611
Weight (kg)	r	−0.166	−0.124	0.505 **	1	0.805 **	−0.378 **	0.016
	*p*	0.073	0.180	0.000		0.000	0.000	0.894
BMI (kg/m^2^)	r	−0.512 **	0.155	−0.091	0.805 **	1	−0.347 **	−0.032
	*p*	0.000	0.094	0.327	0.000		0.000	0.792
Accelerometer-based step counts	r	0.350 **	−0.215 *	−0.130	−0.378 **	−0.347 **	1	0.025
	*p*	0.000	0.020	0.162	0.000	0.000		0.834
App-based step counts	r	0.351 **	−0.021	0.061	0.016	−0.032	0.025	1
	*p*	0.003	0.864	0.611	0.894	0.792	0.834	

* *p* < 0.05; ** *p* < 0.01; BMI: Body Mass Index.

**Table 4:** Predictive model for six-minute walk distance.

Variables	Unstandardized Coefficients		Standardized Coefficients				95% Confidence Interval of B	
	B	Standard Error	Beta	Partial R^2^	ΔR^2^	*p*	Lower Limit	Upper LIMIT
Constant	795.456	593.820				0.000	676.021	914.891
Height_m_ × app-based step counts	0.815	0.121	2.114	0.230	0.230	0.000	0.574	1.056
Age (years)	−1.620	0.754	−0.185	0.348	0.119	0.035	−3.125	−0.115
Weight (kg)	−3.005	0.482	−0.597	0.437	0.088	0.000	−3.967	−2.042
App-based step counts	−1.155	0.214	−1.634	0.609	0.172	0.000	−1.583	−0.727

Model R^2^ = 0.609; Standard error of estimate = 51.3 m. App-based step counts: total number of steps obtained through smartphone app at the end of six-minute walk test.

## Data Availability

The datasets used and/or analyzed during the current study are available from the corresponding author on reasonable request and with permission of Victor Dourado.
